# A critical analysis of the tumour immunosurveillance controversy for 3-MCA-induced sarcomas

**DOI:** 10.1038/sj.bjc.6605198

**Published:** 2009-07-28

**Authors:** T H Schreiber, E R Podack

**Affiliations:** 1 Sheila and David Fuentes Program in Cancer Biology, Sylvester Comprehensive Cancer Center and the Department of Microbiology and Immunology, University of Miami Miller School of Medicine, Miami, FL, USA

**Keywords:** immunosurveillance, immunoediting, methylcholanthrene

## Abstract

The cancer immunoediting hypothesis has gained significant footing over the past decade as a result of work performed using sarcomas induced by 3-methylcholanthrene (3-MCA) in mice. Despite the progress made by several groups in establishing evidence for the three phases of immunoediting (elimination, equilibrium and escape), there continues to be active controversy on the nature of interaction between spontaneously formed tumour cells and the immune system during the early phases of tumourigenesis. At the root of this controversy is conflicting and unresolved evidence spanning back to the 1970s regarding the incidence and frequency of 3-MCA-induced sarcomas in immunocompetent mice as compared to immunodeficient mice. In this mini review we provide a critical analysis of both sides of this controversy.

Tumour immunosurveillance is the process through which the innate and adaptive immune systems identify and eliminate self-cells that have become transformed through spontaneous, chemically or virally induced genetic alterations. The concept of tumour immunosurveillance has endured many false starts over the past century, but the framework first articulated by Burnet and Thomas in the late 1950s (Burnet, 1957), has been supported by numerous reports over the past decade. One of the earliest known studies that linked immune activation to cancer regression was conducted by William Coley beginning in 1893. Later known as ‘Coley's toxin’, the killed mixture of *Streptococcus pyogenes* and *Serratia marcescens* was used in a number of clinical trials up through the 1980s. The most robust of these studies demonstrated clinical benefits for the treatment of hepatocellular carcinoma, nodular lymphoma and various inoperable tumours that failed to reach statistical significance (Johnston and Novales, 1962; Johnston, 1962; Tang *et al*, 1991). The most apparent therapy used today that descended from Coleys toxin is with the use of Bacillus Calmette-Guerin for the treatment of bladder cancer.

The pace of research in understanding tumour/immune interactions slowed significantly in the late 1970s, following a report in *Science* by Osias Stutman (Stutman, 1974). In this report, the rate and frequency of sarcoma formation following chemical induction with 3-methylcholanthrene (3-MCA) was investigated in immunocompetent and athymic mice. The results of this study were devastating, and showed that there was no difference in either the time required for tumours to develop, or the overall tumour frequency, in immunocompetent mice as compared to athymic mice. The observation that immunodeficient mice and immunocompetent mice were equally susceptible to chemically induced tumour formation was a substantial obstacle for Burnet and Thomas’ immunosurveillance hypothesis to overcome. This mini review is written from the sidelines of the controversy surrounding 3-MCA-induced sarcomas. From this vantage point an attempt is made to reveal areas in which the reinterpretation of experimental design and results may guide future studies that will ultimately settle ‘the immunosurveillance controversy’ and fortify the foundation of the ‘immunoediting hypothesis’.

## Clinical and experimental support for tumour immunosurveillance

Clinical data began to emerge in the mid 1980s and through the 1990s both from solid organ and bone marrow transplant recipients and from HIV/AIDS patients that supported a role for the immune system in preventing tumour growth. Early in the onset of the AIDS epidemic, several groups began to report on the high frequency of Kaposi's sarcoma in immunocompromised patients (Pape *et al*, 1983; Haverkos and Drotman, 1985). The incidence of Kaposi's sarcoma among men with AIDS decreased from 40% in 1981 to 20% in 1992, but still remains the most common AIDS-associated cancer in the United States (Biggar and Rabkin, 1996). Kaposi's sarcoma is also associated with immunosuppression following solid organ transplantation, with an overall risk of 0.5% (Farge, 1993). These data support the concept that the immune system can actively suppress the outgrowth of latent tumours, but it is important to note that immune control of virally induced tumours has not been a controversial issue. Other cancers with an increased risk following immunosuppression and organ transplantation include kidney, bladder, malignant melanoma, liver, colon, cervical, prostate, breast, pancreatic, brain, thyroid, bone and connective tissue (Curtis *et al*, 1997; Kasiske *et al*, 2004).

It was not until the mid 1990s that new evidence began to emerge from several laboratories, in additional support to the clinical data that reinvigorated the pursuit of understanding the immune control of cancer. The generation of perforin-1 knockout mice (Kagi *et al*, 1994), led to a series of studies demonstrating that mice with a profound defect in cell-mediated cytotoxicity are more susceptible to chemically induced tumour formation (van den Broek *et al*, 1996), rapid tumour growth (van den Broek *et al*, 1996; Smyth *et al*, 1998) and tumour metastasis (Smyth *et al*, 1999). These reports clearly demonstrated a role for adaptive immunity in the control of cancer, and were coincident with reports in *PNAS* and then *Nature,* by Robert Schreiber's group, that flew directly in the face of the 1974 paper by Osias Stutman (Kaplan *et al*, 1998; Shankaran *et al*, 2001). These experiments substantially advanced the evidence that adaptive immunity helps control tumour formation by showing that in addition to perforin-1, IFN-*γ*, STAT-1 and T cells all contribute to reducing 3-MCA-induced tumour formation and growth. Furthermore, Shankaran *et al*, demonstrated for the first time that tumours which grew in immunodeficient mice were more immunogenic than tumours arising in immunocompetent mice, which fortified Burnet and Thomas’ original theory of immunosurveillance and led to the formal articulation of the immunoediting hypothesis in 2002 (Dunn *et al*, 2002). At the same time tumour-specific antigens were identified in a number of different tumour models, providing for the first time a mechanistic basis for adaptive immune destruction of transformed cells (Van den Eynde and van der Bruggen, 1997; Van Der Bruggen *et al*, 2002). The immunoediting hypothesis consists of three main phases of tumour/immune interactions: elimination (the first phase, during which immunogenic tumour cells are killed by infiltrating immune cells often eliminates all transformed cells, but may occasionally leave a population of cells behind that are less ‘visible’ to the immune system), equilibrium (the second phase, during which the growth of the tumour is roughly equal to the immune system's ability to control it has a variable duration, in some cases spanning the life of the individual) and escape (the final phase, when a combination of reduced immunogenicity, acquisition of immune suppressor mechanisms or through the development of more rapid growth potential the tumour is capable of outpacing an immune response).

## The (lack of) consensus in the literature

Since these reports, the Schreiber group and others have continued to advance the understanding of immunoediting, equilibrium and escape; however, the discrepancies between 3-MCA-induced tumour formation in the Stutman report and in the Shankaran report have yet to be resolved. More importantly, there is a lack of independent reports which specifically address the incidence of 3-MCA-induced sarcomas in immunocompetent and immunodeficient mice, to validate Shankaran *et al*, and the few reports that have been published by investigators attempting to duplicate those data have resulted in a *bona fide* controversy (Engel *et al*, 1997; Qin and Blankenstein, 2004). In a 2004 *Nature* correspondence, Qin and Blankenstein provide evidence that is reminiscent of Stutman's work, demonstrating that the rate and frequency of 3-MCA-induced tumour formation is similar between RAG-1^−/−^ or perforin-1^−/−^ mice and immunocompetent mice. In addition to Stutman and Blankenstein, a report by Noguchi *et al* also found no difference in the incidence of tumours between immunocompetent and immunodeficient mice, although this finding was not emphasised in the report (Noguchi *et al*, 1996). Table 1 provides a summary of published studies examining tumour formation following chemical induction by 3-MCA.

**Figure 1:**
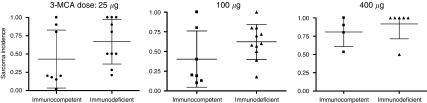
Tumour incidence was plotted for three doses of 3-MCA from the data in Table 1. Strains of mice and routes of injection are not distinguished in this figure. Furthermore, all immunodeficient knockout mouse models displayed in Table 1 are lumped together as ‘immunodeficient’ in this figure. Error bars are ±s.e.m.

After comparing these results, it is first important to note that no two studies can be directly compared because at least one critical parameter (3-MCA dose, injection site or strain of mice used) differs between studies. Individual doses may be compared among the van den Broek, Kaplan and Cretney studies, but this comparison reveals the heterogeneity in tumour formation by identical strains of mice to equal dose and injection protocols of 3-MCA (Kaplan *et al*, 1998; Cretney *et al*, 2002; Table 1; Figure 1). Van den Broek *et al*, Kaplan *et al*, Cretney *et al* and Qin *et al* have all published intrastudy dose response effects for 3-MCA-induced tumour formation (Qin *et al*, 2002). Taken independently, each of these studies demonstrate a 3-MCA dose-dependent increase of sarcoma formation, and in each study the difference in tumour formation between immunocompetent and immunodeficient mice is lost at the highest dose (van den Broek *et al*, 1996; Kaplan *et al*, 1998; Cretney *et al*, 2002; Hayakawa *et al*, 2003). An interstudy comparison obscures these differences (Figure 1). Although these studies all agree that the greatest difference in tumour formation between immunocompetent and immunodeficient mice is observed at the lowest dose (25 *μ*g), one study shows that this difference is completely lost at the intermediate dose (100 *μ*g), whereas another suggests that the difference is actually increased. In C57BL/6 mice, the difference in tumour incidence between wild-type and perforin-1-deficient mice drops from 0.8 with a dose of 25 *μ*g 3-MCA to 0.2 with a dose of 100 *μ*g 3-MCA, whereas in 129/SvEv mice the difference is stable at doses of 25 and 100 *μ*g 3-MCA (0.26 and 0.29, respectively). Shankaran *et al* also used a dose of 100 *μ*g 3-MCA, and reported a difference in tumour incidence of ∼0.4, with even the most severely immunocompromised group forming tumours only 72% of the time. Interestingly, reports from the same laboratory, using the same strains of mice and method of 3-MCA administration demonstrate interexperimental variability of as much as 35% (Kaplan *et al* compared to Shankaran *et al*). This degree of interexperimental variability raises concerns regarding studies that selectively pooled data from several large experiments for multiple publications (Smyth, 2008). The Stutman study also used a dose of 100 *μ*g 3-MCA; however, the strain of mice in this study was CBA/H, and no significant difference in tumour formation was reported between immunocompetent and immunodeficient mice. Two factors that also inhibit direct comparisons between the Stutman study and others are (1) animals were injected with 3-MCA as neonates and (2) the time to follow-up was significantly shorter than other studies. Even studies by the same laboratory, Shankaran *et al* and Koebel *et al* (Koebel *et al*, 2007) or Qin *et al* and Qin and Blankenstein, cannot be directly compared because the two studies differ both in the dose of 3-MCA and the strains of mice used. Value may be derived without comparing, however, given the data illustrating a 3-MCA dose-response, the data suggest that C57BL/6 mice may be substantially more susceptible to 3-MCA-induced tumour formation because 18% of immunocompetent C57BL/6 mice formed progressively growing tumours before day 100, whereas it took immunocompetent 129/SvEv mice >150 days to reach the same incidence with a 4-fold higher dose of 3-MCA (or a 16-fold higher dose for the Blankenstein studies).

In addition to significant differences in tumour formation depending on the dose of 3-MCA and strain of mice used, the route of injection or the use of littermates as controls may also play a significant role. Qin and Blankenstein use an identical dose of 3-MCA and strain of mice to van den Broek *et al*; however, the route of injection varies, intramuscular and subcutaneous, respectively. This suggests that the route of injection may alter tumour formation by as much as 60% in immunocompetent mice. The use of littermates as controls, as well as restriction of comparisons to animals maintained within identical housing parameters, has been suggested as an addition precaution to reduce the observed variability in 3-MCA-induced tumour formation (Blankenstein and Qin, 2003).

## Defining small masses and tumours

Yet another variable that may confound the reported differences between studies and between immunocompetent and immunodeficient mice may arise from reporting the percentage of tumour-free mice compared to reporting tumour growth curves. Stutman, van den Broek, Smyth (Smyth *et al*, 2005), Cretney, Hayakawa, Noguchi, Kaplan and Qin all report percentages of tumour-free mice (or tumour incidence), whereas the two papers from the Schreiber group, Shankaran and Koebel, provide data for individual tumour size over time. Koebel *et al* articulated a potential reason for these differences in their report, providing elegant evidence for the role of adaptive immunity in ‘maintaining occult cancer in an equilibrium state’. These authors consider mice with masses smaller than ∼0.3 cm^2^, ‘tumour-free’. As other authors do not provide evidence of tumour size, it is not possible to determine whether similar parameters were set, or whether small nodules were counted as tumours regardless of size or kinetics of growth. If this ‘tumour-free’ assumption was applied to previous reports on plots showing the percentage of tumour-free mice, it would likely lead to curves for immunocompetent mice with a more gradual slope.

The impact of this definition may be assessed by examining the changes in the results presented by Koebel *et al* if all masses, regardless of size, were scored as tumours. These studies utilised C57BL/6 mice injected subcutaneously with a dose of 25 *μ*g 3-MCA. If mice with masses less than ∼0.3 cm^2^ are scored as ‘tumour-free’, the overall incidence for immunocompetent mice is 0.18 and 0.21 for the two groups followed up to 200 days. If the tumour incidence in the same group of mice is assessed regardless of the size of the mass, the tumour incidence increases from 0.18 to 0.56 and from 0.21 to 0.47 in the two groups of immunocompetent mice. In this study the immunodeficient group of mice were injected with a lower dose (5 *μ*g/ml) of 3-MCA, so direct comparisons cannot be drawn between the immunocompetent and immunodeficient groups. Regardless, the interstudy variability illustrated in Table 1 suggests that the application of a separate analytical filter which alters the tumour incidence between 2- and 3-fold is a potential explanation for otherwise irreconcilable data between investigators.

## Refining the immunoediting hypothesis

The crux of the argument provided by Qin and Blankenstein antagonising immunosurveillance of autochthonous tumours is not that immunosurveillance does not occur, but that subclinical tumours induce immune tolerance, rather than evolving through a process of immunoselection. Although current analysis of the literature falls short of resolving this controversy, it is tempting to speculate that elements of both theories may be true on a case-by-case basis for chemically induced tumours. Due to the heterogeneity of 3-MCA-induced sarcomas in immunocompetent mice, a few being rapidly progressive and the majority developing to a small size and eventually being either rejected or displaying late spontaneous outgrowth, it is highly likely that the immune response to these tumours is also heterogeneous. Rapidly progressive sarcomas may acquire an array of genetic mutations that impart a particularly aggressive phenotype capable of out-pacing an immune response in an immunocompetent animal. These rapidly progressing tumours may also never acquire particularly immunogenic mutations, and therefore remain below the threshold of immune detection. Tumours, which develop slowly in immunocompetent mice and endure a period of ‘equilibrium’ with the immune system may eventually develop sufficient clonal antigenicity to engage an immune response. This immune response may then either lead to elimination of the tumour, or may be overcome by the tumour either via the induction of tolerance, the acquisition of local and/or systemic immunosuppressive elements by the tumour or by an increased proliferative capacity resulting from additional genetic mutations.

Another observation made by Koebel *et al* is the lag time in immunocompetent mice between the emergence of rapidly growing tumours and the times at which ‘stable’ tumours emerge. There appears to be a ‘tumour-free’ period of about 40 days between the rapidly growing tumours and the stable masses. It would be expected that within a given tissue, a specific chemical insult would have a predictable lag time between the insult and the average time at which tumours emerged, and that the emergence of tumours following that lag time would follow a Gaussian distribution. However, the data clearly indicate a bimodal distribution of tumour incidence with an early peak of aggressive tumours and a delayed peak of less-aggressive, immunogenic tumours. Therefore, it is possible that most mice, both immunocompetent and immunodeficient, develop tumours at equivalent time points following a given inoculation with 3-MCA, but that immunocompetent mice are capable of eliminating the majority of transformed cells at this early time point (even before a tumour nodule is palpable), which may or may not reemerge following clonal expansion and enter a period of immune equilibrium.

In contrast to chemically induced tumours, virally induced cancers result from the prolonged expression of viral proteins, many of which are highly immunogenic, and must necessarily evade immune elimination either by the induction of tolerance, the loss of immunogenicity or, as in the case of Kaposi's sarcoma, remain latent until an immunoprivileged opportunity arises (Luppi *et al*, 2000; Scadden, 2003; Kannagi, 2007). Two impressive examples of how viruses can evolve to manipulate the immunogenicity of the cells they infect are KSHV (HHV-8) and HTLV-1. KSHV is able to establish long-term infection of the human host because of the functions of the proteins encoded by the virus. Specifically, KSHV-encoded vFLIP and vIRF-1 enable the virus to differentially regulate MHC-I transcription, allowing the virus to maintain an immune stalemate, which protects both the host and the virus from destruction (Lagos *et al*, 2007). In the case of HTLV-1, the virally encoded Tax protein directs a multitude of intracellular functions promoting the survival and proliferation of infected cells, but it is also a powerful antigen in the evolution of an anti-HTLV-1 immune response; indeed, progression from chronic HTLV-1 infection to adult T-cell leukaemia often coincides with the reduced expression of the Tax protein (Kannagi, 2007).

Autochthonous tumours are inherently different from virally induced tumours in that spontaneous tumours develop mutations over time, which may or may not result in the presentation of an immunogenic peptide. Gene-targeted models of spontaneous tumours have been developed recently, and have begun to illustrate the importance of innate immune surveillance and editing of emerging tumours. Using models of spontaneous prostatic adenocarcinoma and B-cell lymphoma, Guerra *et al* (2008) illustrate that mice deficient in the natural-killer cell receptor, NKG2D, develop earlier and more aggressive disease than the immunocompetent mice. These authors also show that the aggressive tumours developed in NKG2D-deficient mice express higher levels of NKG2D ligands (which mediate NK-mediated destruction of some tumour cells) than the tumour cells from wild-type mice, suggesting that in immunocompetent mice one of the early events in the immunoediting of spontaneous tumours may be the selection of tumour-cell variants that express low levels of NKG2D ligands.

The hypothesis that both spontaneous and virally induced tumours can be recognised and controlled by the immune system is now clearly supported both by clinical and laboratory data (Farge, 1993; Curtis *et al*, 1997; Luppi *et al*, 2000; Scadden, 2003; Kasiske *et al*, 2004; Kannagi, 2007; Lagos *et al*, 2007). Furthermore, the idea that tumour development in an immunocompetent animal proceeds through a variable sequence of immunologic checkpoints involving tolerance induction, immunoselection, immune evasion and/or immune elimination has been well supported by experimentation with 3-MCA-induced sarcomas (van den Broek *et al*, 1996; Kaplan *et al*, 1998; Smyth *et al*, 1999; Shankaran *et al*, 2001; Dunn *et al*, 2002; Koebel *et al*, 2007). The resolution to this controversy will likely require larger scale studies by multiple investigators using identical chemical dosing and delivery protocols, in identical strains of immunocompetent and immunodeficient mice. A major goal of these studies should be to narrow the predicted range of sarcoma formation at individual 3-MCA doses (Figure 1), such that comparisons between immunocompetent and immunodeficient mice may be validated. Given the importance of tumour-specific or -associated antigens in the generation of antitumour immune responses, these studies should also be performed using a system where antigens may be defined and tracked throughout the development of spontaneous or chemically induced tumours, techniques for which have been described (Willimsky *et al*, 2008). The criteria for categorising ‘tumour-free’ mice must also be clarified. Furthermore, recent work with spontaneous tumour models suggests that the role of the innate immune system in controlling tumour formation may be underappreciated by chemically induced models of tumour formation (Guerra *et al*, 2008), so future studies should seek to incorporate elements of both systems to fully appreciate the immune control of cancer. Even after these studies are performed, it is highly likely that some tumours will be found to induce early immune tolerance, some will develop independent of immune regulation and some will run the proposed gauntlet of immune elimination, equilibrium and eventually escape.

## Figures and Tables

**Table 1:**
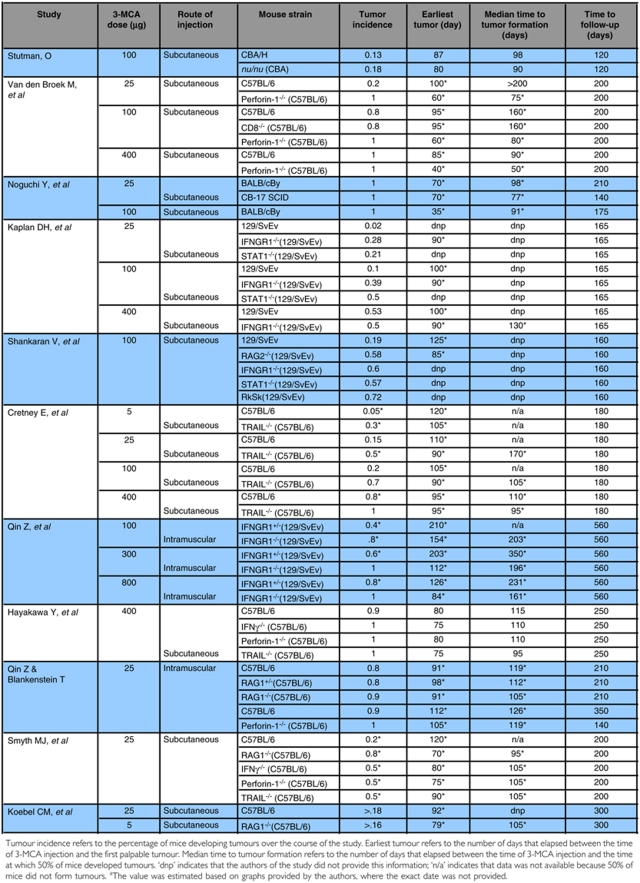
Summary of tumour formation following chemical induction by 3-methylcholanthrene by various studies

## References

[bib1] Biggar RJ, Rabkin CS (1996) The epidemiology of AIDS--related neoplasms. Hematol Oncol Clin North Am 10: 997–1010 8880192 10.1016/s0889-8588(05)70380-4

[bib2] Blankenstein T, Qin Z (2003) Chemical carcinogens as foreign bodies and some pitfalls regarding cancer immune surveillance. Adv Cancer Res 90: 179–207 14710951 10.1016/s0065-230x(03)90006-6

[bib3] Burnet M (1957) Cancer; a biological approach. I. The processes of control. Br Med J 1: 779–786 13404306 10.1136/bmj.1.5022.779 PMC1973174

[bib4] Cretney E, Takeda K, Yagita H, Glaccum M, Peschon JJ, Smyth MJ (2002) Increased susceptibility to tumor initiation and metastasis in TNF-related apoptosis-inducing ligand-deficient mice. J Immunol 168: 1356–1361 11801676 10.4049/jimmunol.168.3.1356

[bib5] Curtis RE, Rowlings PA, Deeg HJ, Shriner DA, Socie G, Travis LB, Horowitz MM, Witherspoon RP, Hoover RN, Sobocinski KA, Fraumeni Jr JF, Boice Jr JD (1997) Solid cancers after bone marrow transplantation. N Engl J Med 336: 897–904 9070469 10.1056/NEJM199703273361301

[bib6] Dunn GP, Bruce AT, Ikeda H, Old LJ, Schreiber RD (2002) Cancer immunoediting: from immunosurveillance to tumor escape. Nat Immunol 3: 991–998 12407406 10.1038/ni1102-991

[bib7] Engel AM, Svane IM, Rygaard J, Werdelin O (1997) MCA sarcomas induced in scid mice are more immunogenic than MCA sarcomas induced in congenic, immunocompetent mice. Scand J Immunol 45: 463–470 9160088 10.1046/j.1365-3083.1997.d01-419.x

[bib8] Farge D (1993) Kaposi's sarcoma in organ transplant recipients. The Collaborative Transplantation Research Group of Ile de France. Eur J Med 2: 339–343 8252179

[bib9] Guerra N, Tan YX, Joncker NT, Choy A, Gallardo F, Xiong N, Knoblaugh S, Cado D, Greenberg NR, Raulet DH (2008) NKG2D-deficient mice are defective in tumor surveillance in models of spontaneous malignancy. Immunity 28: 571–580 18394936 10.1016/j.immuni.2008.02.016 PMC3528789

[bib10] Haverkos HW, Drotman DP (1985) Prevalence of Kaposi's sarcoma among patients with AIDS. N Engl J Med 312: 1518 3990755

[bib11] Hayakawa Y, Rovero S, Forni G, Smyth MJ (2003) Alpha-galactosylceramide (KRN7000) suppression of chemical- and oncogene-dependent carcinogenesis. Proc Natl Acad Sci USA 100: 9464–9469 12867593 10.1073/pnas.1630663100 PMC170941

[bib12] Johnston BJ (1962) Clinical effects of Coley's toxin. I. A controlled study. Cancer Chemother Rep 21: 19–41 14452139

[bib13] Johnston BJ, Novales ET (1962) Clinical effect of Coley's toxin. II. A seven-year study. Cancer Chemother Rep 21: 43–68 14452138

[bib14] Kagi D, Ledermann B, Burki K, Seiler P, Odermatt B, Olsen KJ, Podack ER, Zinkernagel RM, Hengartner H (1994) Cytotoxicity mediated by T cells and natural killer cells is greatly impaired in perforin-deficient mice. Nature 369: 31–37 8164737 10.1038/369031a0

[bib15] Kannagi M (2007) Immunologic control of human T-cell leukemia virus type I and adult T-cell leukemia. Int J Hematol 86: 113–117 17875523 10.1532/IJH97.07092

[bib16] Kaplan DH, Shankaran V, Dighe AS, Stockert E, Aguet M, Old LJ, Schreiber RD (1998) Demonstration of an interferon gamma-dependent tumor surveillance system in immunocompetent mice. Proc Natl Acad Sci USA 95: 7556–7561 9636188 10.1073/pnas.95.13.7556 PMC22681

[bib17] Kasiske BL, Snyder JJ, Gilbertson DT, Wang C (2004) Cancer after kidney transplantation in the United States. Am J Transplant 4: 905–913 15147424 10.1111/j.1600-6143.2004.00450.x

[bib18] Koebel CM, Vermi W, Swann JB, Zerafa N, Rodig SJ, Old LJ, Smyth MJ, Schreiber RD (2007) Adaptive immunity maintains occult cancer in an equilibrium state. Nature 450: 903–907 18026089 10.1038/nature06309

[bib19] Lagos D, Trotter MW, Vart RJ, Wang HW, Matthews NC, Hansen A, Flore O, Gotch F, Boshoff C (2007) Kaposi sarcoma herpesvirus-encoded vFLIP and vIRF1 regulate antigen presentation in lymphatic endothelial cells. Blood 109: 1550–1558 17047149 10.1182/blood-2006-05-024034

[bib20] Luppi M, Barozzi P, Schulz TF, Setti G, Staskus K, Trovato R, Narni F, Donelli A, Maiorana A, Marasca R, Sandrini S, Torelli G (2000) Bone marrow failure associated with human herpesvirus 8 infection after transplantation. N Engl J Med 343: 1378–1385 11070102 10.1056/NEJM200011093431905

[bib21] Noguchi Y, Jungbluth A, Richards EC, Old LJ (1996) Effect of interleukin 12 on tumor induction by 3-methylcholanthrene. Proc Natl Acad Sci USA 93: 11798–11801 8876217 10.1073/pnas.93.21.11798 PMC38138

[bib22] Pape JW, Liautaud B, Thomas F, Mathurin JR, St Amand MM, Boncy M, Pean V, Pamphile M, Laroche AC, Johnson Jr WD (1983) Characteristics of the acquired immunodeficiency syndrome (AIDS) in Haiti. N Engl J Med 309: 945–950 6621622 10.1056/NEJM198310203091603

[bib23] Qin Z, Blankenstein T (2004) A cancer immunosurveillance controversy. Nat Immunol 5: 3–4; author reply 4-5 14699396 10.1038/ni0104-3

[bib24] Qin Z, Kim HJ, Hemme J, Blankenstein T (2002) Inhibition of methylcholanthrene-induced carcinogenesis by an interferon gamma receptor-dependent foreign body reaction. J Exp Med 195: 1479–1490 12045246 10.1084/jem.20011887 PMC2193538

[bib25] Scadden DT (2003) AIDS-related malignancies. Annu Rev Med 54: 285–303 12525676 10.1146/annurev.med.54.101601.152143

[bib26] Shankaran V, Ikeda H, Bruce AT, White JM, Swanson PE, Old LJ, Schreiber RD (2001) IFNgamma and lymphocytes prevent primary tumour development and shape tumour immunogenicity. Nature 410: 1107–1111 11323675 10.1038/35074122

[bib27] Smyth MJ (2008) Clarification of data used in three studies on MCA-induction of sarcoma in mice. Blood 111: 4419 10.1182/blood-2008-02-140103 18398063

[bib28] Smyth MJ, Kelly JM, Baxter AG, Korner H, Sedgwick JD (1998) An essential role for tumor necrosis factor in natural killer cell-mediated tumor rejection in the peritoneum. J Exp Med 188: 1611–1619 9802973 10.1084/jem.188.9.1611 PMC2212521

[bib29] Smyth MJ, Swann J, Cretney E, Zerafa N, Yokoyama WM, Hayakawa Y (2005) NKG2D function protects the host from tumor initiation. J Exp Med 202: 583–588 16129707 10.1084/jem.20050994 PMC2212868

[bib30] Smyth MJ, Thia KY, Cretney E, Kelly JM, Snook MB, Forbes CA, Scalzo AA (1999) Perforin is a major contributor to NK cell control of tumor metastasis. J Immunol 162: 6658–6662 10352283

[bib31] Stutman O (1974) Tumor development after 3-methylcholanthrene in immunologically deficient athymic-nude mice. Science 183: 534–536 4588620 10.1126/science.183.4124.534

[bib32] Tang ZY, Zhou HY, Zhao G, Chai LM, Zhou M, Lu JZ, Liu KD, Havas HF, Nauts HC (1991) Preliminary result of mixed bacterial vaccine as adjuvant treatment of hepatocellular carcinoma. Med Oncol Tumor Pharmacother 8: 23–28 1645825 10.1007/BF02988567

[bib33] van den Broek ME, Kagi D, Ossendorp F, Toes R, Vamvakas S, Lutz WK, Melief CJ, Zinkernagel RM, Hengartner H (1996) Decreased tumor surveillance in perforin-deficient mice. J Exp Med 184: 1781–1790 8920866 10.1084/jem.184.5.1781 PMC2192859

[bib34] Van den Eynde BJ, van der Bruggen P (1997) T cell defined tumor antigens. Curr Opin Immunol 9: 684–693 9368778 10.1016/s0952-7915(97)80050-7

[bib35] Van Der Bruggen P, Zhang Y, Chaux P, Stroobant V, Panichelli C, Schultz ES, Chapiro J, Van Den Eynde BJ, Brasseur F, Boon T (2002) Tumor-specific shared antigenic peptides recognized by human T cells. Immunol Rev 188: 51–64 12445281 10.1034/j.1600-065x.2002.18806.x

[bib36] Willimsky G, Czeh M, Loddenkemper C, Gellermann J, Schmidt K, Wust P, Stein H, Blankenstein T (2008) Immunogenicity of premalignant lesions is the primary cause of general cytotoxic T lymphocyte unresponsiveness. J Exp Med 205: 1687–1700 18573907 10.1084/jem.20072016 PMC2442645

